# Physico-chemical characterization of bilirubin-10-sulfonate and comparison of its acid–base behavior with unconjugated bilirubin

**DOI:** 10.1038/s41598-021-92377-8

**Published:** 2021-06-18

**Authors:** Adam Čepa, Veronika Dejmková, Ladislav Lešetický, Ivan Jelínek, Stanislav Smrček, Martin Štícha, Jana Jašprová, Marie Urbanová, Iryna Goncharova, Martin Dračínský, Eliška Procházková, Donald J. Ostrow, Libor Vítek

**Affiliations:** 1 Department of Organic Chemistry, Faculty of Science, Charles University, Prague, Czech Republic; 2 Institute of Medical Biochemistry and Laboratory Diagnostics, 1st Faculty of Medicine, Charles University, Na Bojišti 3, Praha 2, 12000 Czech Republic; 3 University of Chemistry and Technology, Prague, Czech Republic; 4 Institute of Organic Chemistry and Biochemistry, Czech Academy of Sciences, Prague, Czech Republic; 5 GI/Hepatology Division, University of Washington School of Medicine, Seattle, WA USA; 6 4th Department of Internal Medicine, 1st Faculty of Medicine, Charles University, Prague, Czech Republic

**Keywords:** Biochemistry, Chemical biology, Neuroscience

## Abstract

Unconjugated bilirubin (UCB) is the end-product of heme catabolism in the intravascular compartment. Although beneficial for human health when mildly elevated in the body, when present at greater than a critical threshold concentration, UCB exerts toxic effects that are related to its physico-chemical properties, particularly affecting the central nervous system. The aim of the present study was to characterize bilirubin-10-sulfonate (ranarubin), a naturally occurring bile pigment, including determination of its mixed acidity constants (p*K*_a_^*^). Thanks to the presence of the sulfonic acid moiety, this compound is more polar compared to UCB, which might theoretically solve the problem with an accurate determination of the UCB p*K*_a_^*^ values of its propionic acid carboxylic groups. Bilirubin-10-sulfonate was synthesized by modification of a previously described procedure; and its properties were studied by mass spectrometry (MS), nuclear magnetic resonance (NMR), infrared (IR), and circular dichroism (CD) spectroscopy. Determination of p*K*_a_^*^ values of bilirubin-10-sulfonate and UCB was performed by capillary electrophoresis with low pigment concentrations in polar buffers. The identity of the synthesized bilirubin-10-sulfonate was confirmed by MS, and the pigment was further characterized by NMR, IR, and CD spectroscopy. The p*K*_a_ values of carboxylic acid moieties of bilirubin-10-sulfonate were determined to be 5.02, whereas those of UCB were determined to be 9.01. The physico-chemical properties of bilirubin-10-sulfonate were partially characterized with low p*K*_a_^*^ values compared to those of UCB, indicating that bilirubin-10-sulfonate cannot be used as a surrogate pigment for UCB chemical studies. In addition, using a different methodological approach, the p*K*_a_^*^ values of UCB were found to be in a mildly alkaline region, confirming the conclusions of a recent critical re-evaluation of this specific issue.

## Introduction

**Figure 1:**
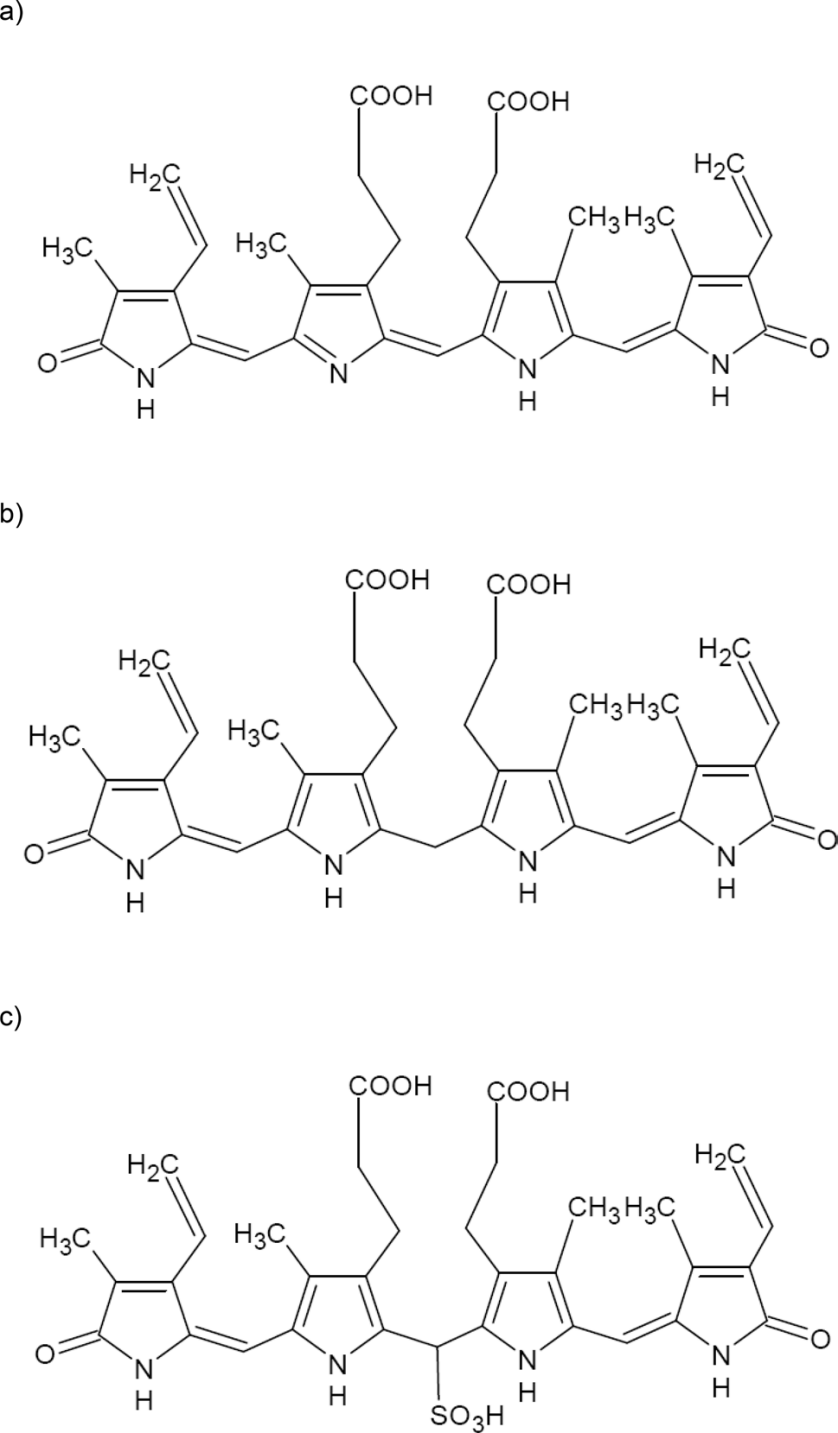
Structures of (**a**) biliverdin, (**b**) unconjugated bilirubin, and (**c**) bilirubin-10-sulfonate (ranarubin).

Unconjugated bilirubin (UCB) (Fig. 1) is the end-product of heme catabolism in the intravascular compartment. Due to its unusual conformation, UCB is a highly lipophilic compound, which when it exceeds a safe threshold level, such as in severe neonatal jaundice, can cross the blood–brain barrier and cause irreversible neurotoxicity^1^. Although it is evident that this process depends on the physico-chemical properties of UCB and its interaction with specific affinity molecules, a proper and detailed explanation of bilirubin transport into the central nervous system still remains to be fully elucidated. The major obstacle in solving the problem of bilirubin's behavior in a water environment, such as blood plasma, is its lipophilicity due to the ridge-tile conformation in which both UCB propionic acid carboxylic groups are entrapped by hydrogen bonds within the molecules; and where only the non-polar functional groups are exposed to the external environment^2^. Many attempts have been made to determine the p*K*_a_ values for both of the propionic acid carboxylic groups; however, with inconclusive results, specifically due to the different conditions used for the determinations of the ionization constants of the carboxyl groups. Indeed, a wide range of p*K*_a_ values have been reported for UCB; with some investigators favoring a p*K*_a_ of 6.2 to 6.5^3^, yet with others suggesting that the true p*K*_a_ values are even higher (6.8 to 9.3) because of intramolecular hydrogen bonding^4,5^.

Ranarubin is the C10-sulfonic acid bilirubin physiologically present in the American bullfrog, *Rana catesbeiana*^6^ (Fig. 1). Thanks to the presence of the sulfonic acid moiety, this bile pigment is more polar compared to UCB, which might theoretically solve the problem with an accurate determination of the UCB p*K*_a_ values.

Therefore, the aim of the present study was to characterize bilirubin-10-sulfonate (ranarubin), including determination of its dissociation constants using a capillary electrophoresis method, as well as to try to use this method for the determination of the dissociation constants of UCB. Capillary electrophoresis was used because it is a valuable and highly developed technique for the determination of dissociation constants of weak acids, bases, and zwitterions, whose primary basis is the experimentally measured dependence of effective electrophoretic mobility on pH. Thus, it provides detailed information about acid–base equilibria after suitable processing expressed as mixed acidity constants, p*K*_a_^*^^7–12^.

## Material and methods

### Chemicals

All chemicals were from Sigma (MO, USA), except for UCB and biliverdin hydrochloride (both from Frontier Scientific, UT, USA). Before use, UCB was purified and re-crystalized as described previously^13^.

### Bilirubin-10-sulfonate synthesis

The bilirubin-10-sulfonate was synthesized by modification of the procedure described in Ma et al*.*^14^. Briefly, biliverdin hydrochloride (Fig. 1) (13.4 mg) was dissolved in 2 mL butanol, and the vial was desiccated by argon gas to prevent oxidation. A water solution of NaHSO_3_ (0.2 mmol/L) was then added to the sample, and the solution was mixed for 30 min at laboratory temperature. An additional 0.1 mL of NaHSO_3_ solution was added to the solution formed, and the final mixture was then mixed for 60 min at laboratory temperature. Then, 0.75 g of Na_2_SO_4_ was added, and the sample was dried for 15 min. The final solution was filtered and precipitated with diethylether. The vials were centrifuged for 20 min at 1500*g*. The supernatant was aspirated, and the precipitate was dried by a stream of argon. The bilirubin-10-sulfonate was then freeze-dried overnight.

### Confirmation of bilirubin-10-sulfonate identity

The ESI–MS spectra to assess the identity and purity of bilirubin-10-sulfonate were acquired on an Esquire 3000 ion trap instrument (Bruker Daltonic, Germany) controlled by Esquire Control 5.3.11 software. The analyses were conducted in negative ion mode using a scan range from 50 to 800 Da, and nitrogen was used as the nebulizer gas at a pressure of 8 psi and a flow rate of 4 L · min^−1^ for the dry gas. The capillary voltage and temperature were set at 4000 V and 300 °C, respectively. The sample solutions were delivered to the nebulizer by a syringe pump (Cole Parmer, USA). NMR spectra were measured on a Bruker AVANCE II 600 MHz spectrometer (^1^H at 600 MHz, ^13^C at 151 MHz) in DMSO-*d*_6_. The spectra were referenced to the residual solvent signals (^1^H at 2.50 ppm, ^13^C at 39.70 ppm). The NMR signals were assigned by a combination of 1D and 2D (H,C-HSQC, and H,C-HMBC) NMR experiments. In addition, NMR spectra of UCB dissolved in DMSO-*d*_6_ and in CDCl_3_ were also recorded on the same instrument. These ^1^H signals were observed:

UCB in DMSO ^1^H NMR (600.13 MHz, DMSO-*d*_6_) 11.92 (bs, 2H, OH), 10.51 (bs, 1H, NH), 10.48 (bs, 1H, NH), 10.10 (bs, 1H, NH), 9.97 (bs, 1H, NH), 6.82 (dd, 1H, *J*_vic_ = 17.6 and 11.7, H-18α), 6.58 (dd, 1H, *J*_vic_ = 17.5 and 11.5, H-3α), 6.20 (dd, 1H, *J*_vic_ = 17.5, *J*_gem_ = 2.8, H-3β*c*), 6.09 (bs, 2H, H-5 and H-15), 5.61–5.65 (m, 2H, H-18β), 5.29 (dd, 1H, *J*_vic_ = 11.6, *J*_gem_ = 2.8, H-3β*t*), 3.97 (s, 2H, H10), 2.43 (m, 4H, CH_2_–CH_2_-COOH), 2.16 (s, 3H, CH_3_), 2.03 (s, 3H, CH_3_), 2.00 (s, 3H, CH_3_), 1.95 (m, 4H, CH_2_-COOH) 1.92 (s, 3H, CH_3_).

UCB in CDCl_3_^1^H NMR (600.13 MHz, CDCl_3_) 13.69 (bs, 2H, OH), 10.80 (bs, 1H, NH), 10.69 (bs, 1H, NH), 9.29 (bs, 1H, NH), 9.26 (bs, 1H, NH), 6.61 (dd, 1H, *J*_vic_ = 17.6 and 11.8, H-18α), 6.49 (dd, 1H, *J*_vic_ = 17.6 and 11.5, H-3α), 6.21 (s, 1H, H-5), 6.16 (dd, 1H, *J*_vic_ = 17.6, *J*_gem_ = 2.2, H-3β*c*), 6.13 (s, 1H, H-15), 5.58–5.62 (m, 2H, H-18β), 5.36 (dd, 1H, *J*_vic_ = 11.5, *J*_gem_ = 2.2, H-3β*t*), 4.08 (s, 2H, H10), 2.78–3.04 (m, 6H, CH_2_–CH_2_-COOH), 2.58 (m, 2H, CH_2_-COOH), 2.18 (s, 3H, CH_3_), 2.16 (s, 3H, CH_3_), 2.15 (s, 3H, CH_3_), 1.99 (s, 3H, CH_3_).

Bilirubin-10-sulfonate in DMSO ^1^H NMR (600.13 MHz, DMSO-*d*_6_) 9.93–10.10 (m, 4H, NH), 6.83 (dd, 1H, *J*_vic_ = 18.2 and 11.1, H-18α), 6.57 (dd, 1H, *J*_vic_ = 17.5 and 11.5, H-3α), 6.19 (dd, 1H, *J*_vic_ = 17.5, *J*_gem_ = 2.8, H-3β*c*), 6.14 and 6.14 (2 × s, 2H, H-5 and H-15), 5.63–5.66 (m, 2H, H-18β), 5.49 (bs, H10), 5.29 (dd, 1H, *J*_vic_ = 11.6, *J*_gem_ = 2.8, H-3β*t*), 2.17 (s, 3H, CH_3_), 2.07 (s, 3H, CH_3_), 2.03 (s, 3H, CH_3_), 1.92 (s, 3H, CH_3_).

### Circular dichroism spectroscopy

The CD spectra of bilirubin-10-sulfonate were measured in a quartz cuvette with an optical path length of 1 cm (Starna, USA) using a J-810 spectropolarimeter (Jasco, Japan). The final spectrum was obtained as the average of 3 accumulations. The spectra were corrected for the baseline by subtracting the spectra of the corresponding solvents. The CD measurements were conducted at room temperature (25 °C). For the spectral measurements, bilirubin-10-sulfonate was used at a concentration of 1.2 × 10^–5^ mol · L^−1^. Human serum albumin (HSA) and bovine serum albumin (BSA) were used as chiral discriminators for bilirubin-10-sulfonate at the pigment/serum albumin molar ratio of 1:1. The FTIR spectrum of bilirubin-10-sulfonate was recorded in the DMSO-d_6_ solution at a concentration of 0.044 mol · L^−1^, with a resolution of 4 · cm^−1^, using an IFS-66/S Fourier transform infrared spectrometer (Bruker, Germany). A de-mountable cell with CaF_2_ windows and a Teflon spacer with a 50-μm pathlength was used.

### Capillary electrophoresis

The determinations of the bilirubin-10-sulfonate and UCB p*K*_a_ values were performed by capillary electrophoresis using an laboratory-made instrument with the following parameters and assay conditions: fused silica capillary (internal diameter, i.d. = 75 μm; outer diameter, o.d. = 375 μm; capillary length to detector, L_d_ = 0.6 m; total capillary length, L_t_ = 0.75 m), 20 kV voltage, injection pressure 20 mbar, injection time 6 s, and photometric detection at λ = 205 nm. Although the separation capillary was not positioned in a thermostated compartment, its temperature was monitored using a miniature thermistor glued on a polyimide surface. The thermal stability of a capillary surface was improved by a gentle and stable flow of argon gas (all experiments were carried out under an argon atmosphere). An increase of the temperature never exceeded 0.5 °C throughout the measurements. Depending on the experiment type, the bilirubin-10-sulfonate was either dissolved in 5% DMSO in deionized water with a final concentration of 0.005–0.1 g/L (3.3–66.3 μmol/L); or to eliminate the role of DMSO on the p*K*_a_ values of the studied bile pigments, low concentrations of both pigments (UCB concentration = 2.9 μmol/L, bilirubin-10-sulfonate concentration = 0.66 μmol/L,) were prepared by dissolving them in the respective background electrolytes, or directly in deionized water. The characteristics of the specific background electrolytes are given in Table 1.

**Table 1:** Background electrolytes used for capillary electrophoresis.

Background electrolyte	Composition	pH
**(a) Bilirubin-10-sulfonate (ranarubin) (c = 66.3 μmol/L, 0.1 g/L)**		
1	30 mM acetic acid + NaOH, DMSO 5%	3.08
2	30 mM acetic acid + NaOH, DMSO 5%	4.23
3	30 mM acetic acid + NaOH, DMSO 5%	4.72
4	30 mM acetic acid + NaOH, DMSO 5%	4.87
5	20 mM phosphoric acid + NaOH	5.05
6	20 mM phosphoric acid + NaOH	5.16
7	20 mM phosphoric acid + NaOH	5.34
8	20 mM phosphoric acid + NaOH	5.61
9	20 mM phosphoric acid + NaOH	6.08
10	20 mM phosphoric acid + NaOH	7.05
11	20 mM boric acid + NaOH	8.22
12	20 mM boric acid + NaOH	9.21
13	10 mM boric acid + NaOH	10.40
**(b) bilirubin-10-sulfonate (ranarubin) (c = 0.663 μmol/L, 0.001 g/L)**		
1	30 mM acetic acid + NaOH	4.21
2	30 mM acetic acid + NaOH	4.77
3	20 mM phosphoric acid + NaOH	5.41
4	20 mM phosphoric acid + NaOH	6.09
5	20 mM phosphoric acid + NaOH	7.05
6	20 mM Na tetraborate + Na dihydrogenphosphate	8.27
**(c) UCB (c = 2.92 μmol/L, 0.005 g/L)**		
1	20 mM Na tetraborate + Na dihydrogenphosphate	8.32
2	20 mM Na tetraborate + Na dihydrogenphosphate	8.52
3	20 mM Na tetraborate + Na dihydrogenphosphate	8.87
4	20 mM Na tetraborate + Na dihydrogenphosphate	9.02
5	20 mM Na tetraborate + Na dihydrogenphosphate	9.13
6	20 mM Na tetraborate	9.27
7	20 mM Na tetraborate + NaOH	9.51
8	20 mM Na tetraborate + NaOH	9.60

The values of effective electrophoretic mobilities, m_eff_ [m^2^/Vs] were calculated from experimental values of migration time, t_m_ [s], and time of electroosmotic marker, t_eo_ [s], according to the equation: $m_{{eff}}  = \frac{{L_{d} L_{t} }}{U}\left( {\frac{1}{{t_{m} }} - \frac{1}{{t_{{eo}} }}} \right)$ where L_d_ [m] and L_t_ [m] is the length of the separation capillary to the detector and its total length, and U is the separation voltage [V]. The m_eff_ vs. pH mobility dependencies was fitted using Boltzmann sigmoidal function $y = \frac{{A2 + (A1 - A2)}}{{1 + e^{{\frac{{x - x_{0} }}{{dx}}}} }}$, where A1 and A2 correspond to minimum and maximum fitted m_eff_ values, and x_0_ is the point of inflection.

For the estimate of mixed acidity constants, p*K*_a_^*^, we adapted the following simple procedure. The average values of m_eff_ calculated from three consecutive measurements at a given pH were taken to construct the m_eff_/pH dependencies. These were fitted with the Boltzmann sigmoidal function employing a standard non-linear fitting procedure provided by Origin 16 software. The calculated point of inflection corresponds to the desired value of p*K*_a_^*^. The measurements were performed at 20 °C and the estimated mixed acidity constants p*K*_a_^*^ are related to this temperature.

Mass spectrometry and capillary electrophoresis analyses proved that components of used background electrolytes did not form complexes or adducts with bilirubin derivatives.

## Results

### Characterization of bilirubin-10-sulfonate

**Figure 2:**
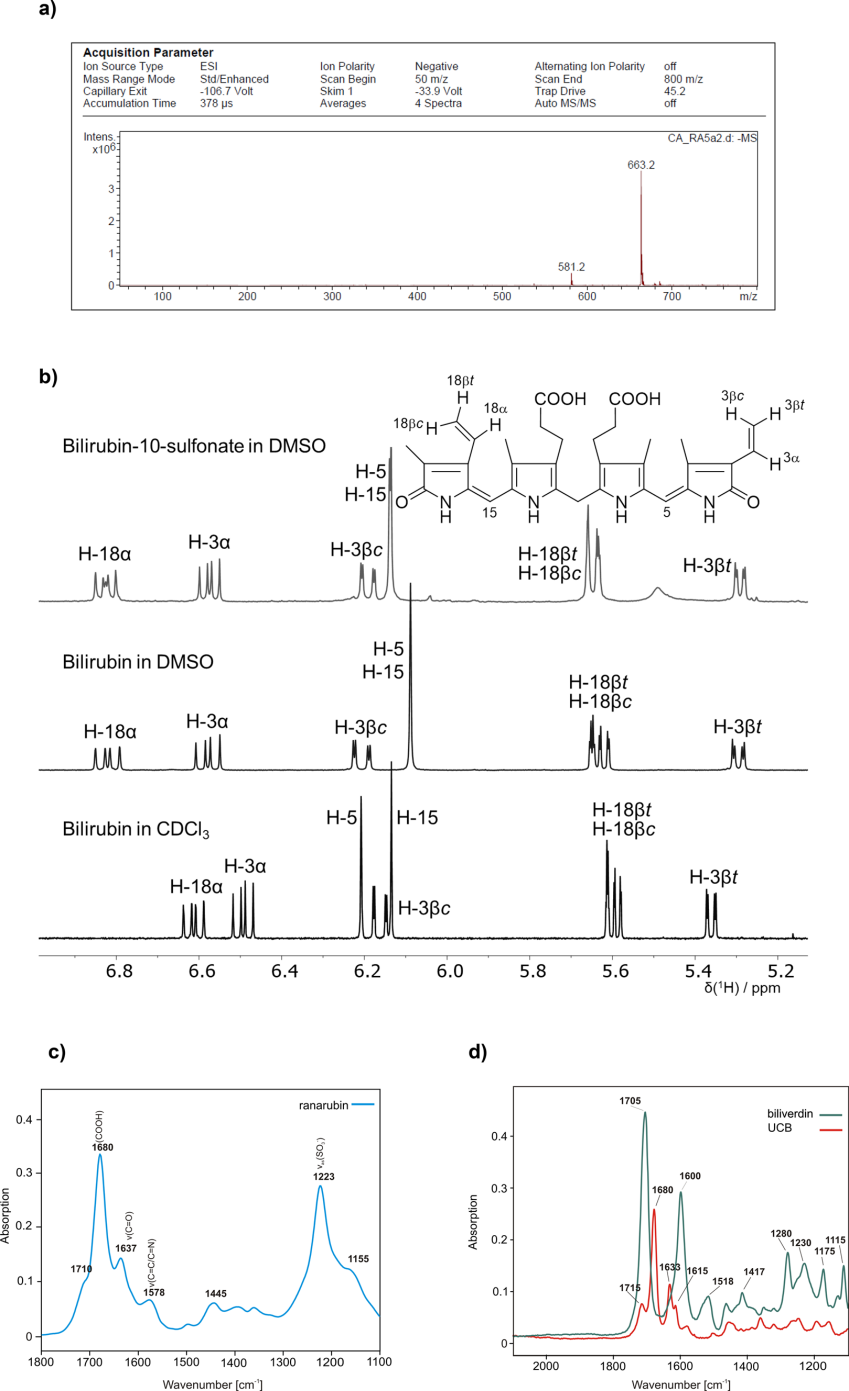
(**a**) Mass spectra of synthesized bilirubin-10-sulfonate (ranarubin); (**b**) The double-bond region of ^1^H NMR spectra of UCB and bilirubin-10-sulfonate; IR spectra in DMSO of (**c**) bilirubin-10-sulfonate (c = 0.044 mol/L, pathlength = 50 μm); and (**d**) biliverdin and UCB (0.075 mol/L, pathlength = 25 μm).

The synthetic procedure used in our studies led to a 75% yield of bilirubin-10-sulfonate, whose identity and purity was confirmed by mass spectrometry (Fig. 2a) as well as by NMR spectroscopy (Fig. 2b). In order to get insights into the conformational behaviors of bilirubin-10-sulfonate and UCB, we compared the ^1^H NMR spectrum of bilirubin-10-sulfonate (measured in DMSO) with those of UCB measured in DMSO and CDCl_3_ (Fig. 2b). The spectrum of UCB in non-polar CDCl_3_ was significantly different from that obtained in DMSO, which can be explained by a substantial conformational change. In chloroform, UCB probably adopts the previously described “closed” ridge-tail conformation. On the other hand, in DMSO that substantially solvates the polar groups of UCB, the conformation is probably “open”, with close contacts between the solvent and both the NH and OH groups of UCB. The double-bond region of the ^1^H spectrum of bilirubin-10-sulfonate is almost identical to that of UCB in DMSO, which indicates that the conformations of both compounds are very similar in this solvent. The NMR spectra of UCB in DMSO are almost identical to those published in Biological Magnetic Resonance Data Bank (https://bmrb.io/metabolomics/mol_summary/jmol_display.php?bmrbid=bmse000627).

In addition, the IR spectra of bilirubin-10-sulfonate corresponded to its structure (Fig. 2c); the IR spectra of bilirubin and biliverdin are given in Fig. 2d for comparison. As can be seen from Fig. 2c,d, the spectra corresponding to C=O vibrations in the bilirubin-10-sulfonate molecule are shifted equally as in the UCB molecule, suggestive of the similar involvement of the C=O group in the intramolecular H bonding. Thus, the conformation of bilirubin-10-sulfonate resembles that of UCB (having six intramolecular H bonds) more than that of biliverdin (with only two intramolecular H bonds).

**Figure 3:**
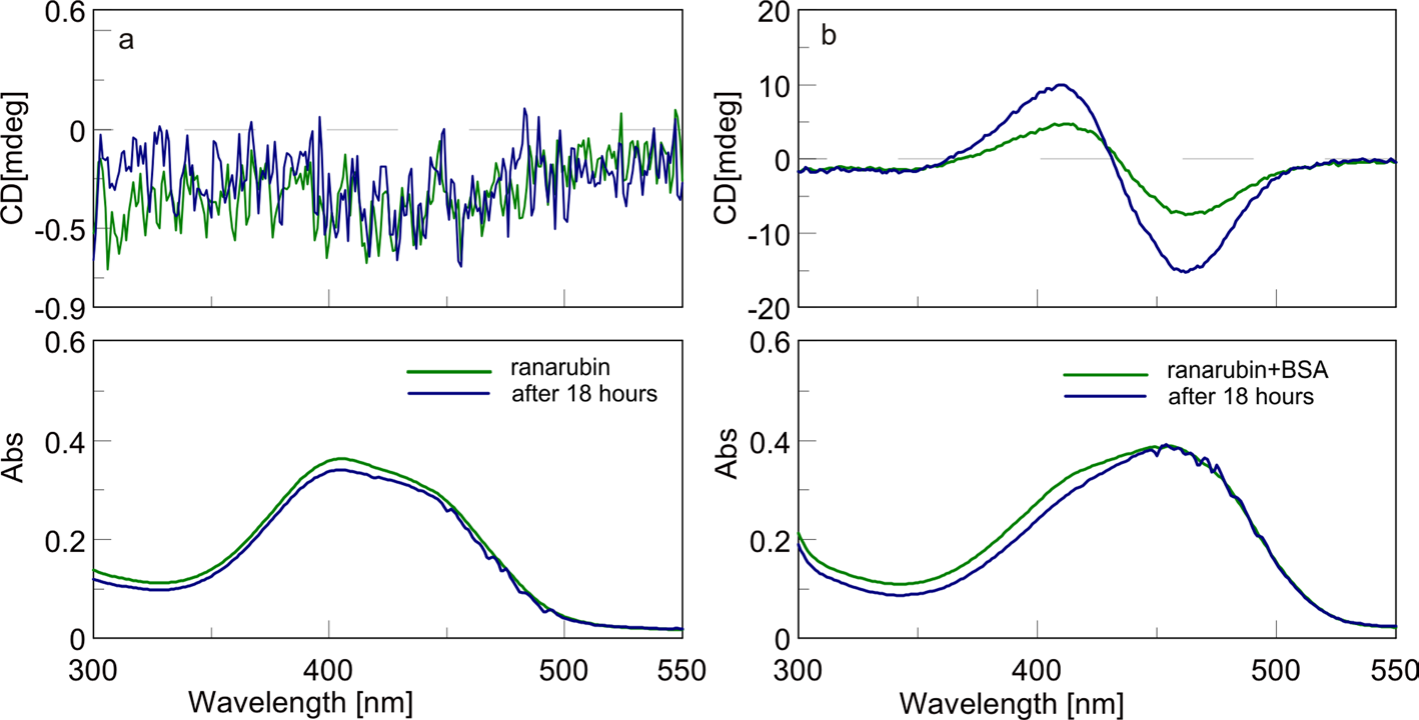
Spectral stability of: (**a**) bilirubin-10-sulfonate (ranarubin) in H_2_O, and (**b**) bilirubin-10-sulfonate (ranarubin) in H_2_O with BSA. *BSA* bovine serum albumin. Bilirubin-10-sulfonate:BSA = 1:1. Measured after 18 h.

The CD experiment revealed that an aqueous solution of bilirubin-10-sulfonate does not exhibit a CD signal, in accordance with its non-enantioselective synthesis. No substantial change in UV absorption was seen within 18 h, indicating that bilirubin-10-sulfonate's stability was sufficient for spectral measurement within several hours (Fig. 3a). In the presence of BSA, a pronounced CD signal in the form of a negative couplet within 18 h reveals preferential enantioselective binding of bilirubin-10-sulfonate to serum albumin (Fig. 3b).

**Figure 4:**
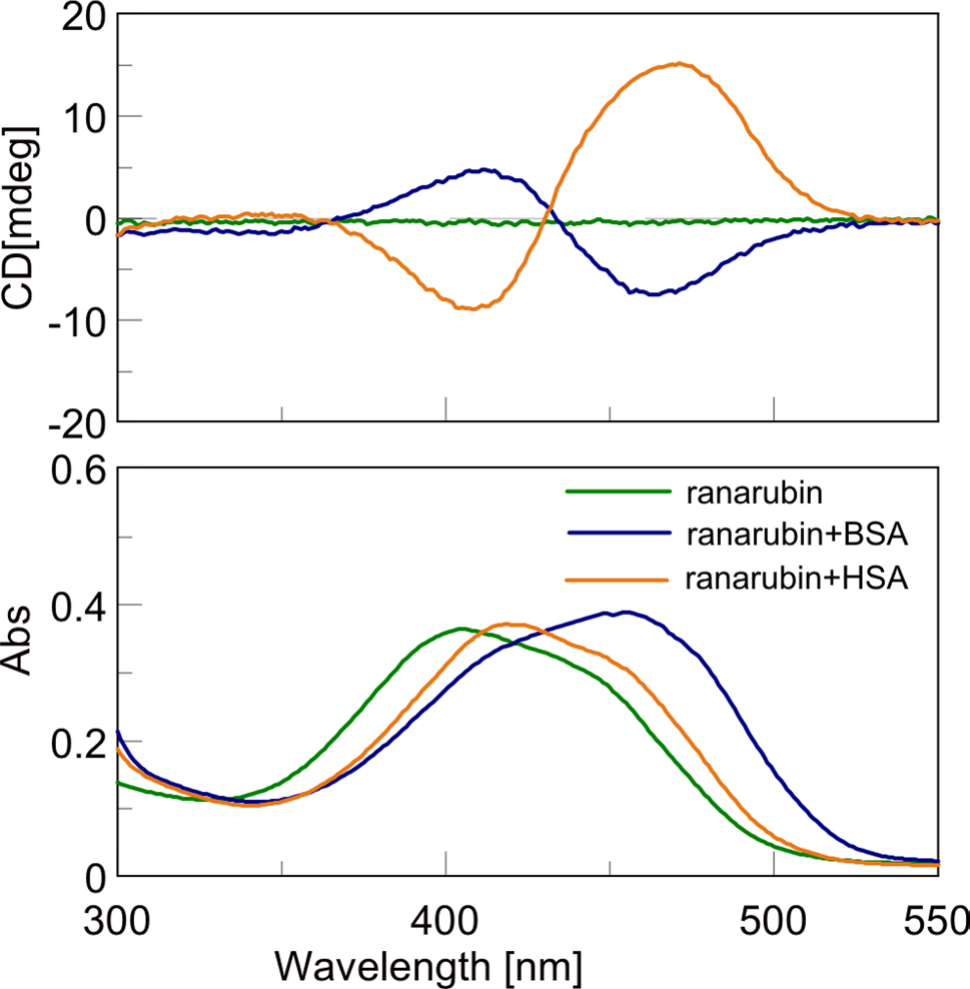
Electronic CD and absorption spectra of bilirubin-10-sulfonate (ranarubin). Bilirubin-10-sulfonate was measured in a mixture with BSA or HSA. Bilirubin-10-sulfonate:serum albumin (molar concentration) = 1:1, measured in v H_2_O, concentration of bilirubin-10-sulfonate = 1.2 × 10^–5^ mol/L.

Electronic CD spectra of bilirubin-10-sulfonate with BSA and HSA (Fig. 4) demonstrated that BSA recognizes bilirubin-10-sulfonate in the *M* conformation; whereas in the *P* conformation for HSA. These spectra, which resemble those of bilirubin, indicate that both chromophores bind to HSA in very similar ways^15,16^.

### Determination of p*K*_a_^*^ values of bilirubin-10-sulfonate and UCB

**Figure 5:**
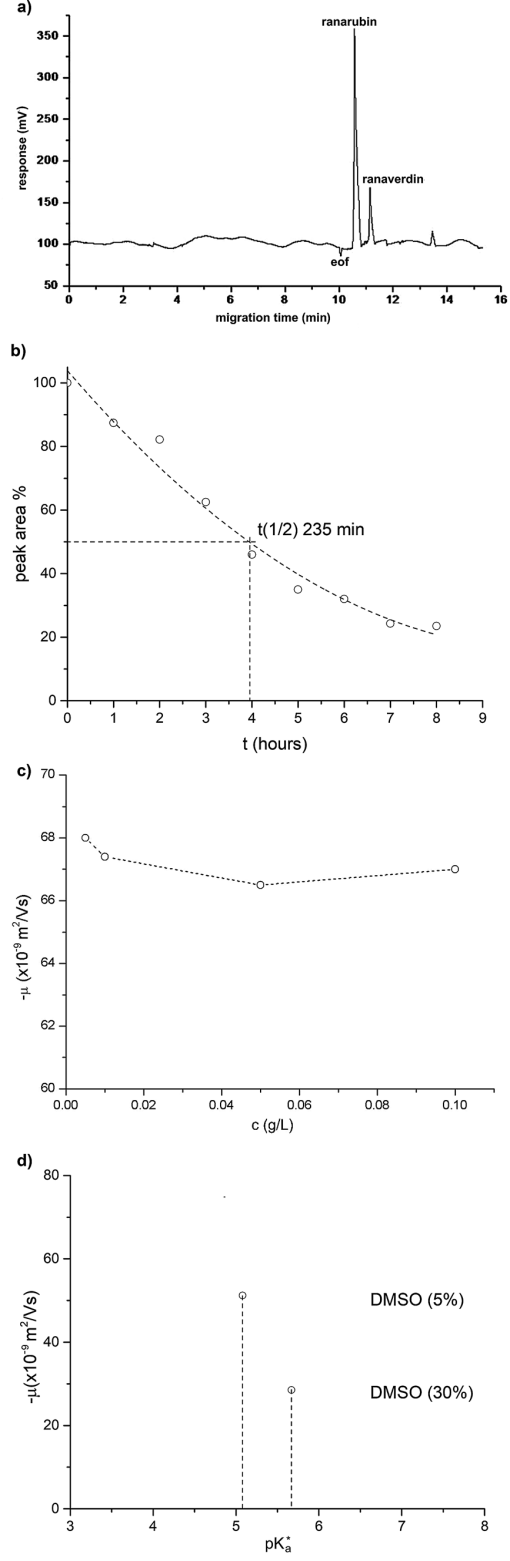
(**a**) Electrophoregram of bilirubin-10-sulfonate (ranarubin) and biliverdin-10-sulfonate (ranaverdin) mixture. Electrophoresis performed in background electrolyte 4 (see Table 1a). Bilirubin-10-sulfonate concentration = 0.1 g/L. *eof* electroosmotic flow rate marker. (**b**) Stability of bilirubin-10-sulfonate. Measured in background electrolyte 5 (see Table 1a). Bilirubin-10-sulfonate concentration = 0.1 g/L. (**c**) Dependence of effective electrophoretic mobility of bilirubin-10-sulfonate on sample concentration. Background electrolyte 12 (see Table 1a). Sample: bilirubin-10-sulfonate 0.1, 0.05, 0.01, 0.005 g/L in deionized water (5% DMSO). (**d**) Dependence of effective electrophoretic mobility and p*K*_a_ value of bilirubin-10-sulfonate on the content of DMSO. Background electrolyte 4 (see Table 1a).

The majority of experiments were performed with bilirubin-10-sulfonate at concentrations of 0.1 g/L (66 μmol/L) dissolved in a mixture of 5% DMSO in deionized water. Electrophoresis under the conditions used clearly separated bilirubin-10-sulfonate from biliverdin-10-sulfonate (ranaverdin, a minor by-product formed during ranarubin synthesis), and also proved its purity (Fig. 5a). The stability of bilirubin-10-sulfonate was fully sufficient for our electrophoresis studies with bilirubin-10-sulfonate half-life in the used background electrolytes of about 6 h (Fig. 5b). Electrophoretic mobility was not dependent on the sample concentration through a wide concentration range (Fig. 5c); but higher percentages of DMSO in the mixture led to increases of the apparent bilirubin-10-sulfonate p*K*_a_^*^ values (Fig. 5d).

**Figure 6:**
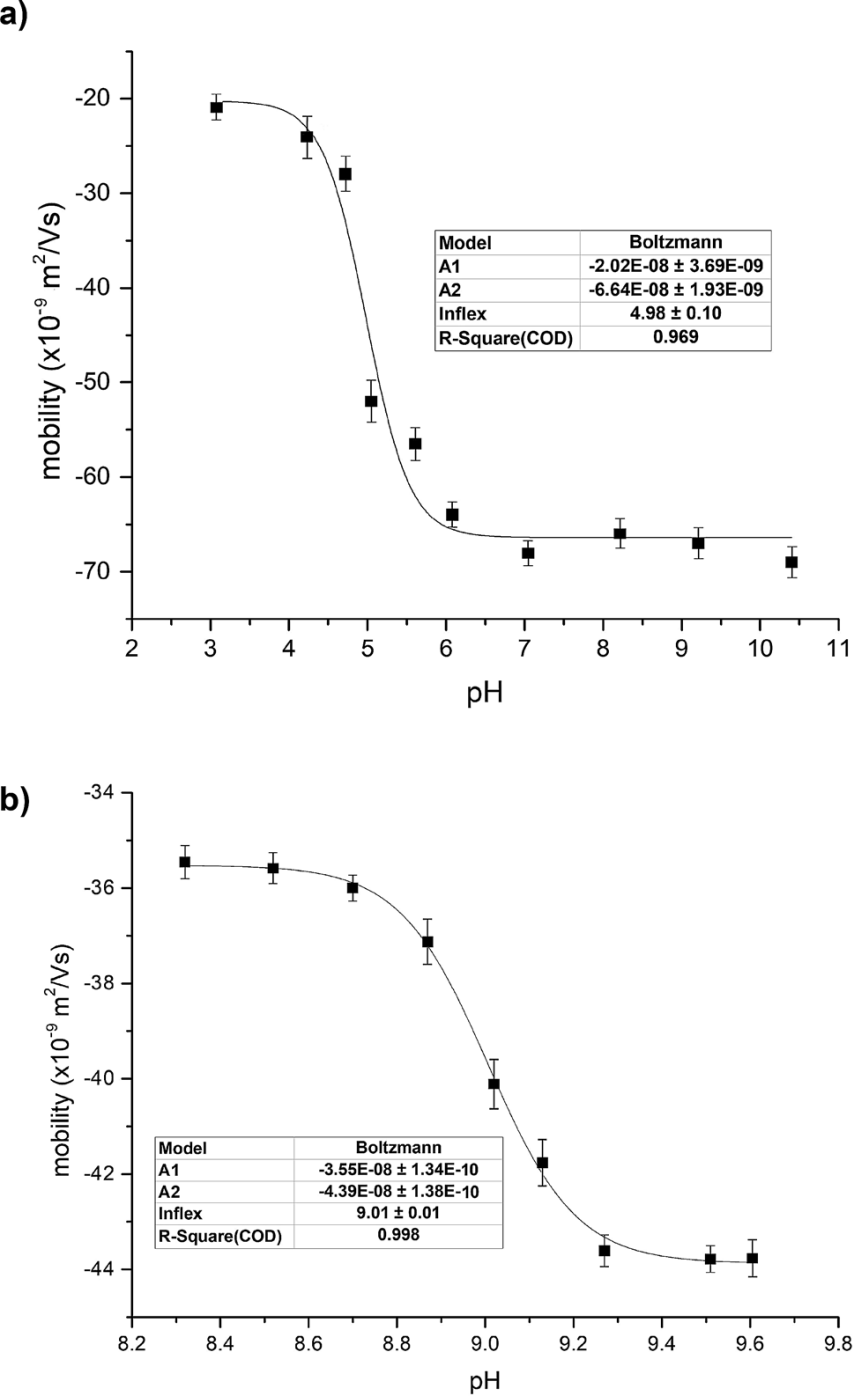
Dependence of effective electrophoretic mobility of (**a**) bilirubin-10-sulfonate (ranarubin) and (**b**) UCB. Background electrolytes 1–3, 6, 9–13 were used for the bilirubin-10-sulfonate studies; background electrolytes 3–5, 7, 8 for the UCB studies. Depicted points are the mean ± SD values of three experimental values. The position of the inflection points was determined using the numerical interpolation procedure using the standard Boltzmann sigmoidal function defined in the Origin software (Origin (Pro), Version 2016, OriginLab Corporation, Northampton, MA, USA Corporation, MA USA).

Using a combination of background electrolytes, the p*K*_a_^*^ values of bilirubin-10-sulfonate dissolved in a 5% DMSO mixture with deionized water were determined to be slightly above 5 (5.02; Fig. 6a). To directly compare the p*K*_a_^*^ values of bilirubin-10-sulfonate with those of UCB we used the same approach as for bilirubin-10-sulfonate analysis; i.e., strictly without DMSO and using only a low concentration of UCB (2.9 μM). Under these conditions, the p*K*_a_^*^ value of UCB was found to be 9.01, thus much higher when compared to that of bilirubin-10-sulfonate (Fig. 6b).

Although the used phosphate buffers possess lower buffering capacity in the weak acidic region, this fact did not affect our results as can be implied from the mobility curves with apparent sigmoidal function and acceptable bias and variance of experimental points. In addition, extended dispersion of separated zones and/or the formation of system peaks were not observed during analyses, indicating reliability of the whole electrophoretic system.

## Discussion

The ionization constants of both carboxylic groups of UCB still remain an unsettled issue, with inconclusive results from previous reports that were mostly due to improper methodological conditions used in these studies. Indeed, p*K*_a_ values ranging from 4.4 to 9.3 (for review see^17^) have been described in the literature. Since most of the carboxylic acid p*K*_a_ values are around 5, and values above 6 are seldom encountered^18,19^, higher p*K*_a_ values reported in some studies suggest the remarkable effect of intramolecular hydrogen bonding on proton dissociation^4,5,20^. Thus, it is not surprising that when using solvents that break the internal hydrogen bonds (such as dimethyl sulfoxide), very low and identical p*K*_a_ values of 4.4 for both carboxyl groups were found^21^. Similar results were observed by Lee et al*.*^22^ with dimethyl formamide, achieving calculated p*K*_a_ values of 4.3 and 5.3 for the two carboxyl groups, respectively. Low p*K*_a_ values (4.2 and 4.9, respectively) were suggested for UCB based on ^13^C-NMR of mesobilibrubin XIIIα in dimethylsulfoxide^18^, but these studies cannot be considered as relevant for a highly polar aqueous environment such as blood plasma (for review see^17^).

Potentiometric titration of bilirubin dianion salt in simple aqueous solution led to progressive precipitation of insoluble bilirubin diacid as the pH fell below about 8.0^20,23^. It has been suggested that the aqueous titration curves could be reconstructed by using the p*K*_a_ values obtained in dimethyl formamide or dimethyl sulfoxide, and applying a mathematical model that assumes that bilirubin is only solubilized by prior ionization of the solid bilirubin diacid. Both potentiometric and spectrophotometric titrations of UCB dissolved in aqueous taurocholate solutions yielded p*K*_a_ values of 6.2–6.5^3^. Even much higher p*K*_a_ values (8.1 and 8.4) were reported in further studies by Hahm et al*.*^20^ for aqueous UCB at physiological ionic strength.

Ranarubin, the C10-sulfonic acid bilirubin used in our study, is probably a more biologically important bile pigment than has previously been considered. Apart from being the major bile pigment of the American bullfrog *Rana catesbeiana*^6^, it is also likely to be present in the lumen of the human gastrointestinal tract, where it is produced from biliverdin by the enteric bacteria *Citrobacter youngae*^24^. This pigment, similar to other known linear tetrapyrroles, has important biological properties and is implicated in protection against oxidative (and also potentially in inflammatory) processes^24,25^.

In our study, bilirubin-10-sulfonate was used to analyze p*K*_a_^*^ values and to compare them with those of UCB using the capillary electrophoresis method. Several important observations were made. Under the conditions used, the sulfonic group of ranarubin was fully dissociated (as implied from the anionic mobility of bilirubin-10-sulfonate in the acidic region of the mobility curve in Fig. 6a); thus, the p*K*_a_^*^ value determined belonged to one of the carboxylic groups of bilirubin-10-sulfonate. However, this value was surprisingly low (at around 5.0). Although they might be different, we were not able to determine the p*K*_a_^*^ value of the 2nd carboxylic group by the method used. However, we presumed the acid–base proximity or even equivalence of both carboxyl groups in the bilirubin-10-sulfonate molecule. Hence, the estimated p*K*_a_^*^ value is an average of two near values of partial dissociation constants. The equality of both carboxyl groups was also confirmed by the shape of the mobility curve with a single inflection point. The low p*K*_a_^*^ value was likely not due to the solvent used, since similar values were found in our experiments with DMSO as well as with polar solvents. The self-aggregation of bilirubin-10-sulfonate also did not seem to contribute to our observations, since similar p*K*_a_^*^ values were also found for low concentrations of bilirubin-10-sulfonate (< 1 μM)^26^. Also, any interactions of bilirubin-10-sulfonate with the capillary wall seem highly unlikely due to the inertness of the capillary materials; additionally, the measured p*K*_a_^*^ value did not belong to its degradation product(s), as evidenced by our studies demonstrating a sufficient stability of bilirubin-10-sulfonate during the performed capillary electrophoresis analyses. The p*K*_a_^*^ value of bilirubin-10-sulfonate observed in our study is lower, but still in accordance with the 6.32 value of the unpublished bilirubin-10-sulfonate potentiometric titration studies performed by Uwaya^27^.

Interestingly, the p*K*_a_^*^ value of UCB did substantially differ from that of bilirubin-10-sulfonate, and it was in the alkaline region, supporting previous data by some other authors^4,5^. Our data are also consistent with the recent observation of Berman and Carey, who described similar pH relationships of UCB in model bile systems, with implications for pigment gallstone formation^28^. In addition, similar conclusions were made by Mukerjee and Ostrow in their comprehensive paper reviewing available data on p*K*_a_ values of bilirubin in non-aqueous media^17^. The low p*K*_a_^*^ value of bilirubin-10-sulfonate seems to be due to its high polarity caused by the sulfonate anion, probably resulting in a p*K*_a_^*^ value that is close to that of short chain carboxylic acids^19^. The difference in p*K*_a_^*^ values of bilirubin-10-sulfonate and UCB may explain the different biological behavior of bilirubin-10-sulfonate with surprisingly strong intraperitoneal absorption and intravascular retention after intravenous or intraperitoneal administration, and little absorption after intraduodenal administration^29^.

In conclusion, the physico-chemical properties of bilirubin-10-sulfonate were partially characterized, with low p*K*_a_^*^ values compared to UCB, indicating that bilirubin-10-sulfonate cannot be used as a surrogate pigment for UCB chemical studies. In addition, using a different methodological approach, the p*K*_a_^*^ values of UCB were found to be in a mild alkaline region, confirming the conclusions of a recent critical re-evaluation of this specific issue.
